# Extracts and Abstracts

**Published:** 1889-01

**Authors:** 


### EXTRACTS AND ABSTRACTS.

***Illumination of the Larynx and other Body Cavities by Transmitted Light.***—At the meeting of the Medical Section of the Schlesischen Gesellschaft fur Vaterlandische Cultur, at Breslau, October 26th and November 9th, Professor Voltolini read a paper upon this subject. We make a few extracts from the report in the *Wiener Med. Wochenschr.*, Nos. 47, 48, 49, of this year:

"I not only hope but am convinced that this new method of examination, which I present to-day, will sooner or later come into common use, and will be of great service in distinguishing benign from malignant growths in the larynx, and will also be of value in physiological investigations.

"This new method consists in the illumination of the larynx—and other body cavities—by transmitted light. In the larynx the light is directed through the tissues of the neck, while the laryngoscopic mirror is passed into the unilluminated pharynx.

"By this means not only are the separate parts of the larynx illuminated and visible as by the usual method, but the whole larynx is illuminated through and through. Everything can be examined. The vocal cords are perfectly transparent and the slightest abnormality upon the surface or within the tissue can be readily seen. In one sense it is possible to examine the tissues in the living body as with a microscope—since often in microscopic examination the main object is to see into the tissues by means of transmitted light. . . . . As above stated this method is especially valuable for the recognition and differentiation of benign and malignant growths in the larynx.

"Growths frequently occur in the ventricles of Morgagni; these cannot be recognized by the usual laryngoscopic methods until they protrude from the ventricles; by means of transmitted light they can be seen when still entirely within the ventricle.

"At the present time Waldeyer's views upon new growths are generally accepted, viz: that in cancer epithelial prolongations grow into the underlying tissue,—briefly, that in malignant tumors the growth extends into the healthy tissues, while in benign tumors the growth is outwards. I have not now a case of cancer of the larynx under observation, but have several cases of benign polypoid growths upon the vocal cords, and I can assure you that the vocal bands appear clear and transparent and the polypi grow outward from the mucous membrane into the lumen of the larynx.

"The illumination of the larynx by transmitted light was first suggested and tried by Czermak, the inventor of the laryngoscope, and endorsed by Gerhardt and Stoerk. Czermak said ('Der Kehlkopfspeigel,' 2d edition, Leipsic, 1863, p. 31): 'I believe that in this method of illumination I have discovered a means of observing changes in the vertical thickness of the vocal cords, both physiological (register of the voice) and pathological, and that it will be a means of examining the deeper tissues directly. The usefulness of this method in rhinoscopy is very limited.'

"In reference to the last sentence I wish to say that the method proposed by me is of very great service in rhinoscopy.

"Soon after Czermak's publication Semeleder discouraged further studies in this direction. In 'Die Laryngoskie,' Vienna, 1863, p. 25, he says: 'This method of illumination seems very plausible, but I do not think it is capable of practical application; for in cases where the examination of the air passages is easy, a much better view is obtained by reflected light, and in cases where obstructions are present it will be fully as difficult to obtain a reflected image of the parts illuminated by transmitted light as by reflected light. Moreover, transmitted light is never sufficiently intense to afford a satisfactory view of the details.'

"Semeleder seems to have misunderstood the principle involved—and not to have tried the method himself; for illumination from above does not give the same definition as that given by transmitted light—just as under the microscope reflected and transmitted light give entirely different images, the one showing us only the surface, the other the structure within the tissue. Further, it is often possible, with transmitted light to obtain a good image of a larynx where it would be impossible to see anything by reflected light. The author demonstrated this point in the case of a boy with enormously enlarged tonsils. A good view of the larynx was obtained though the tonsils nearly hid the mirror—just as any cavity lighted from within can be fully viewed through an opening much too small to admit sufficient light for its illumination. Since Semeleder's publication, his method has scarcely been mentioned by any writer upon laryngology, although Professor Voltolini has regularly demonstrated it to his students.

"Heretofore the use of sunlight only has been practicable for this purpose, and this is the chief reason why the method has not grown in favor. For the sun is not always shining, and we are limited in our opportunities for making examination. Moreover, direct sunlight is not sufficiently intense; it must be concentrated upon the bared skin of the neck at the risk of burning it. The difficulties in manipulation are also considerable: The light must be directed upon different sides of the throat successively; any movement during the examination on the part of the patient or of the condensing mirror leaves the larynx dark. The position of the patient, with the mouth open and the chin depressed, is a further disadvantage, and if the patient wears a chin beard, and if in addition to this it is necessary to draw the tongue forward, holding it with a napkin, the obstacles are multiplied, and great expertness is needed to obviate them.

"With the discovery of the incandescent lamp by Edison, this method of illumination has taken a different form. We have now an adequate light that can be used at any time, and upon all patients.

"The lamp for this purpose is prepared as follows: The back of the lamp is silvered to serve as a reflector. A small shoemaker's globe, filled with cold water, is adjusted in front of the lamp and serves the double purpose of concentrating the light and cutting off the heat. The lamp and ball are held in a metal case. For use it is adjustable upon the neck, and the observer is free to make any manipulation or to put the patient in any position he desires."

In a case demonstrated before the society, the whole larynx was perfectly illuminated, and the pharynx even, was partially illuminated from top to bottom.

A smaller lamp that can be held in the mouth, is used for illuminating the tissues and bones of the face. It is described more fully in the author's work on "Diseases of the Nose," just published.

Professor Voltolini uses three styles of lamps; a large one, 4 cm. in diameter, arranged as above described, for illuminating the larynx; one of medium size, to be held in the mouth, for illuminating the face; and a small lamp for introduction into the naso-pharyngeal space. He predicts that this method of illumination will be used in examining other cavities of the body, and mentions particularly the stomach and the uterus. F. S. J.

***Curability of Cancerous Disease.***—Dr. Edward von Meyer has a contribution to this subject in *Deutsche Zeitschrift für Chirurgie*, Bd. 28, Hft. 1 and 2.

Taking all the cases that show a definite duration of cure, 22, it is seen that carcinoma affected 11 cases, or 50 per cent., sarcoma 7 cases, cysto-sarcoma 2 cases, and melano-sarcoma and mixed tumor 1 case each. Of the cured cases of carcinoma there were: 1 case of carcinoma mammæ, 3 cases of genital carcinoma, 2 of nasal, 2 of labial, and 1 each of inferior maxillary, trunk, and extremity carcinoma.

The cases of cured sarcoma were: 1 of the genital apparatus, 1 of the nose, 2 of the lower jaw, and 1 each of the trunk, extremities, and head.

The 2 cases of cured cysto-sarcoma were of the mamma and lower jaw.

The longest duration of cure was in the cases of sarcoma, cysto-sarcoma, and fibro-sarcoma. Of the cases of carcinoma, 7 showed a duration of cure of more than 3 years, and 4 a cure of less than 3 years before the occurrence of death from other causes. In none of these cases were the neighboring lymphatics implicated. None of these cases had recurrence. In one case only, a spindle-celled sarcoma of the thigh, was it necessary to operate after 3 years for recurrence in the mammary gland.

Following is a résumé of all the cases mentioned: Of the 64 cases, 47 were carcinoma, 11 sarcoma, 3 cysto-sarcoma, 1 each of melano-sarcoma, fibro-sarcoma, and carcinoma-sarcomatodes. Of these, 41 were cured of the cancerous diseases, 22 being still living, and 19 have died of other affections. Of the patients now living, 7 have lived from 17 years 7 months, to 20 years 3 months; 2 are living after 17 years 4 months; 5 after 15 years to 17 years 3 months; and 8 from 9 years 7 months to 13 years 7 months. Only 1 person has lived less than 10 years 5 months. Of the 19 that have died without recurrence, 6 lived 10 years or more; 6 from 6 years 5

months to 8 years; 5 from 2 years to 4½ years; and 2 as much as 1½ years.

The nature of the tumors in those still living has been given. Of the 19 that died free of recurrence, 14 had carcinoma, 3 sarcoma, and 1 each cysto-sarcoma and fibro-sarcoma.

The regions affected by the tumors were as follows: *Carcinoma:* of the mamma 4, nose 6, lip 5, lower jaw and trunk 1 each, extremities 2, genital apparatus 5, head 1. *Sarcoma:* 1 each of the mamma, nose, trunk, genitals. head, and upper jaw; and 2 each of the lower jaw and extremities. *Cysto-sarcoma:* mamma 2, lower jaw 1. *Melano-sarcoma:* 1 of trunk. *Carcinoma-sarcomatodes:* 1 of extremities. *Fibro-sarcoma:* 1 of head.

The regional lymphatics were involved in 2 cases of carcinoma and 2 of sarcoma.

Removal of recurrence after removal of primary tumors (37 cases): 1 recurrence in 1 case (melano-sarcoma of inguinal glands); 2 recurrences in 1 case (carcinoma of the lip); 3 in 1 case (cysto-sarcoma of lower jaw); 4 recurrences in 1 case (sarcoma of lower jaw).

Of the 18 that died of cancerous disease, 9 died of local recurrence; 4 of metastasis; 3 of metastasis and local recurrence; 2 of impossibility of operation.

Recurrence of carcinoma took place as follows: nose, lower lip and cheek, mamma, forehead, lower jaw, 1 case each; upper jaw, 2 cases. Sarcoma recurred in one case on the mamma.

Metastasis affected the liver in three cases, after carcinoma of the leg, mamma, and maxilla; the stomach in one case, after carcinoma of the lower lip.

Metastasis and local recurrence took place in 2 cases of carcinoma mammæ, the metastasis being to the abdomen and leg, and to the liver; and in 1 case of perineal carcinoma, the metastasis being to the glands.

Recurrence took place usually within a year, in 1 case only later than 2 years.

The inoperable case was one of œsophageal carcinoma.

The duration of life of those that died of recurrence or metastasis was 11½ years in 1 case; 10½ in 1; 5¾ in 1; 5 in 1; 1½ in 2; and 1¼, 1, 8 months and 3 months in 1 case each.

Meyer has no doubt that the results would show still better had it been possible to follow up the private cases, of which a large number must be free of recurrence.

---

***Curative Erysipelas.***—For some time erysipelas has been known to have a modifying or curative influence on chronic skin diseases. SCHWIMMER has seen variable results in cases of syphilitic disease of the skin. Sometimes the erysipelas causes the secondary symptoms to disappear for a time, and sometimes it permanently cures grave syphilitic affections, such as serpiginous syphilides. Schwimmer has had a case in which eczema of the face and tumefaction of the cervical glands were cured by an attack of erysipelas. In another case an orchi-epididymitis, which had remained obstinate for some time, passed away suddenly during an attack of facial erysipelas. In still another case keloid growths, developed in the cicatrices of burns, were removed by erysipelas.—*Archives Générales de Médicine*, Dec., 1888.

---

***Faradism in Hysteria and Epilepsy.***—DR. A. DIDIER, in an article on the use of faradization as a means of arresting hysterical attacks and of diagnosticating between hysteria and epilepsy, draws the following conclusions:

1. Faradization is the best means of arresting or preventing an hysterical attack. It will abort cases of convulsive hysteria and some cases of hystero-epilepsy.

2. In difficult cases this method of electrization enables one to distinguish between an epileptic and a person with an attack of hysteria, or a case of epileptiform hysteria, or the convulsive hysteria of Charcot; the faradization has no influence on the epileptic attack.

3. In case of a person attacked by epilepsy and hysteria at the same time, the hysterical symptoms will disappear under faradization.

4. The method of using this kind of electrization consists in applying the electrodes of a faradic current of medium intensity along the path of the aura, that is, in the epigastric space and to the anterior portion of the neck, at the beginning of the attack. When the patient is not seen until the tonic or clonic period, one electrode should be placed on the neck and the other in one hand; or both electrodes may be placed in the hands.

5. Besides its abortive effects on the hysterical crisis, faradization has a curative effect, as has been known for some time.—*Lyon Médical*, No. 49, 1888.

---

***Surgical Treatment of Suppurating Venereal Buboes.***—In the *Vierteljahreschrift für Dermatologie und Syphiligraphie*, Heft 2, 1888, is a paper giving Fleischer and Szadek's results of the treatment of suppurating buboes in the Military Hospital at Kiew, according to the method of Fleischer, introduced in 1880.

The total number of cases of bubo treated was 274, occurring in 1,084 cases of soft chancre treated in 1882-86. These figures seem to show that bubo develops in about one case in four of soft chancre, but Szadek says that it is not the extent of the chancre, but its seat and virulence that determine the inflammation of the glands. The bubo formed sometimes when the sore was healing, and in some cases even after cicatrization. Most frequently complicated with bubo were sores of the frænum and on the inner surface of the prepuce, and the majority of these buboes suppurated. Rarely was the suppuration limited to a single gland in these cases, and in almost all the abscesses several glands were involved, and soon became connected with the original opening by sinuses.

In 105 cases the bubo was situated in the right groin, in the left in 131, and both groins were affected in 38 cases. In the majority of cases of double bubo the chancre was on the frænum.

After the development of adenitis the chancre healed quickly. In most cases the bubo appeared three or four weeks after the chancre, but in some cases it did not appear until two weeks after the chancre had healed. From the beginning to the opening of the bubo in healthy persons was as a rule from two three weeks; but in anæmic, scrofulous, and tuberculous patients it was as long as from four to eight weeks.

The treatment of these cases was conducted on the following plan: In the earlier stage, so long as there was no fluctuation or redness of the skin, the only means used was rest and avoidance of all inguinal irritation, with antiseptic treatment of the chancre. If the skin was red, but fluctutation was not present over the whole swelling, hot compresses steeped in carbolic lotion were applied till uniform and complete suppuration occurred. Attempts were made in many cases to cause absorption of the bubo, as by painting it with tincture of iodine; but these attempts all failed, the only result being some relief to pain and a longer course of the abscess. When fluctuation was complete, the hair was removed, the skin thoroughly cleansed with soap, followed by the application of a 5 per cent. solution of carbolic acid, or a 1 per cent. solution of corrosive sublimate. Then an incision, corresponding with the length of the bubo, was usually made parallel to Poupart's ligament, the pus was evacuated, any existing sinuses laid open with scissors, and all swollen glands removed by means of the fingers or sharp spoon, the capsule of the gland being incised when necessary. Lastly, all destroyed portions of skin were removed by means of the scissors. The abscess-cavity was now washed out with corrosive sublimate solution, powdered with iodoform or iodoform and alum, and filled with iodoform gauze. Over this was a dressing composed of layers of gauze or wool impregnated with sublimate and salicylic wool was applied, and then a ball of jute or tow, and the whole covered with a piece of macintosh or varnished paper, and secured by a spica bandage. This dressing was generally left for from two to five days, when the wound was simply dressed with iodoform, the second dressing being left until oozing appeared at the edges.

The wound usually healed under from two to five dressings, the average duration being 30 days. No burrowing nor formation of pus occurred, and in no case did the buboes become chancrous. Erysipelas occurred in 5 cases, and eczema in the neighborhood of the wound 12 times; 26 suppurating syphilitic buboes were opened, and healed without scraping or removal of glands; 12 cases of tuberculous adenitis of the groin were treated by removal of glands, scraping, and application of iodoform. There were no cases of iodoform poisoning.

---

*Dyspeptic Dyspnœa.*—Dr. W. H. Katzenbach says that dyspeptic dyspnœa is characterized by a sense of weight or oppression across the chest, an almost constant desire to draw a long breath, and a feeling that the air does not enter the lungs to a sufficient depth. Repeated inspiratory efforts are made. The body is fixed in some certain position, or a sudden quick change of position is made before a satisfactory inspiration is obtained, but the relief is of short duration, and these efforts must be soon repeated. Diversion, intense occupation, or active bodily exercise, such as rapid walking, sometimes mitigates or relieves the attack. None of these ever aggravates it. Dyspeptic dyspnœa occurs after meals, in the intervals of stomach digestion, and not infrequently at night. The duration of the attacks is variable, but they usually extend over a considerable portion of the day or night. They usually recur daily, or at intervals of a few days, for weeks or months, or there may be but a single attack.

There is a more alarming form of dyspeptic dyspnœa, the paroxysms of which occur at night only. Patients complain of severe attacks of shortness of breath, of smothering coming on at night, and unaccompanied by wheezing, as in asthma. The paroxysm usually occurs after the patient has gone to bed. He is awakened with a feeling of oppression and weight across the chest, a distressing sense of suffocation, and inability to get his breath. He sits up in bed, and makes strong efforts to inspire, but the air does not seem to enter his lungs. No position gives comfort. Becoming excited, his heart beats rapidly, and this increases his distress and discomfort. The respiration is slow, irregular and sighing. The expression is one of great suffering. The face is pale, the surface cool, and perspiration may appear on the forehead only, or upon the whole body. The heart's action is usually rapid and irregular; it may be slow and regular.

The attack lasts from half an hour to an hour or more. It subsides gradually, and the patient falls asleep, but he may be awakened by another before morning. The attacks may recur several successive nights, or at longer intervals over a considerable period.

The ordinary mild form of dyspeptic dyspnœa is to be distinguished from the dyspnœa of anæmia, chlorosis, or hysteria. In anæmia the breathing is rapid while the individual is quiet, though he may not be conscious of it, but it is still more rapid, labored and suffocative from effort or excitement. The more severe and nocturnal form of dyspeptic dyspnœa is to be distinguished from that occurring in uræmia. In the latter the difficulty of breathing is very distressing, but the patient is less excited. He gasps for breath quietly, but with great effort. Here, also, are symptoms of organic disease. In bronchial asthma the dyspnœa is expiratory, the breathing is audible at a distance, and signs of bronchial spasm are obtained by auscultation. In the dyspnœa from cardiac disease the respiration is panting, and cyanosis and anasarca, with other symptoms of heart disease, are present.

The majority of cases of dyspeptic dyspnœa yield readily to treatment directed to the morbid conditions present in the digestive system. If the attacks of dyspeptic dyspnœa are of the milder form, and occur in the intervals of stomach digestion, measures must be employed to improve the functions of the digestive organs and restore nutrition to a healthy state. Diet, clothing, bathing, hours for rest and sleep, as well as for exercise and diversion, must be regulated. The abuse of alcohol, tobacco, coffee, tea and venery must be corrected. Electricity, massage and sea bathing are valuable agents in treating the chronic dyspeptic. If the patient suffering with a severe attack of dyspeptic dyspnœa has recently taken food, the stomach should be evacuated, by an emetic or the stomach tube, and further washed out. This should be followed by tonics and aromatics.—*N. Y. Medical Journal*, Dec. 29, 1888.

---

***Spontaneous Strangulation of the Limbs.***—Under the name *ainhum* Dr. Silva first described in 1886, in the *Gaceta Medica de Bahia*, a curious spontaneous strangulation of the limbs. Dr. C. Buicli, of Bucharest, records two cases in the *Archives Roumaines de Médicine et de Chirurgie*, No. 1. From previously recorded cases, and from his two, Buicli concludes:

1. That the process by virtue of which the strangulation becomes operative, takes place during intra-uterine life. This process becomes completely developed before birth. No change takes place afterwards in the state of the parts.

2. The constricting furrow is sometimes simple and isolated, sometimes accompanied by congenital vices of conformation which lead to a true mutilation of the limbs. It is in the latter cases situated preferably at the level of the smaller members—the fingers; the lesion is bounded by simple circular depressions, or is characterized by the absence of one or more phalanges.

3. At the place of constriction there is sometimes obnubilation of sensibility; in other cases this change is absent.

4. The depression affects not only the skin and soft parts, but also the deep tissues, the bones.

5. While this lesion may be congenital it does not appear to be hereditary.

---

***Medical Department, U. S. Navy.***—Surgeon-General J. M. Browne has recently submitted the report on the health and general condition of the Navy, so far as concerns the Bureau of Medicine and Surgery.

The Naval hospitals are reported as being generally in a satisfactory condition, but the hospitals at Portsmouth, N. H., and Norfolk, Va., are in a dilapidated condition.

The Museum of Hygiene is overcrowded, and should have a much larger building. At present it has only a part of a building. Our Government seems to be excessively one-sided in the matter of liberality. This is seen in the case of the vacancies in the Medical corps of the Navy. The condition of assistant surgeons in the Navy, in the matters of rank and pay, is such that it is difficult to get desirable men to apply for the service.

Passed Assistant Surgeon C. W. Rush makes an interesting report on the medical condition of Alaska, the inhabitants and diseases of the country, and the mineral springs, of which there are a great many. These springs are hot and cold, and impregnated with sulphur, iron, and magnesia.

Passed Assistant Surgeon F. S. Nash has a report on the expedition to Northern Alaska. His report is unfavorable to the use of lime-juice as an antiscorbutic.

Medical Inspector T. C. Walton reports an epidemic of "sore throat" at U. S. Naval Academy at Annapolis; the sore throat was of diphtheritic nature. The most satisfactory general plan of treatment was to begin with a saline cathartic; give liquor ammon. acetatis during the height of the febrile stage; then tinct. ferri muriatis in from twelve to thirty drop doses, diluted with glycerine, every two to four hours, according to the degree of lessened vitality; also alcohol rather freely, as milk-punch or whisky and water; hot milk, beef-tea, and ice when asked for. Locally, frequent spraying of the throat with lime-water, applying tinct. ferri muriatis, pure or diluted one-half with glycerine, over the affected parts every three or four hours; syringing the throat and nostrils daily with a solution of chlorinated soda, or more frequently when fœtor was perceptible, care being taken not to forcibly detach the membrane. Externally over the painful glands a mixture of tincture of belladonna and iodine afforded relief. The patients were isolated until recovery was complete.

From the report on the Museum of Hygiene we learn that Medical Inspector Charles H. White has devised a simple method for the determination of urea, the apparatus for which can be easily made and used on board ship. Unfortunately, the method is not described in the report.

---

***Aspiration and Drainage in Purulent Pericarditis.***—At the meeting of the Clinical Society of London on Nov. 23, Dr. Dickinson related the case of a boy, aged 10, who was brought to St. George's Hospital with symptoms supposed to be pyæmic. A large gluteal abscess was followed by signs of pleural effusion and œdema of the face and chest. On admission, June 15, 1887, there was evidence of effusion in the left pleura and in the pericardium. The position of the heart was almost undiscoverable amid the dullness, which involved the left pleural and præcordial regions. There was much dyspnœa, blueness, and irregularity of the pulse. Œdema was more or less general, but especially marked about the thorax. The liver was enlarged or depressed so as to reach the umbilicus.

On June 18 the pleura was aspirated, and 37 ounces of serum were drawn off: this operation was repeated on the 23d with the removal of 32 ounces. The dyspnœa, blueness and œdema were but slightly and temporarily relieved by the operation, which was repeated on June 25 and 28, so rapid was the accumulation and so great the distress.

On June 30, the futility of dealing with the pleura having become apparent, the pericardium was aspirated, and one ounce of creamy pus withdrawn. On July 8 the aspiration was repeated with more success, 12 ounces of creamy pus being evacuated, and on the 15th with the removal of 19 ounces. The puncture was made on the right side close to the edge of the sternum, in the 5th interspace. Before each of these operations the heart was carried to the left by an evacuation of the pleura. The lower part of the pericardium, where the swing of the heart was greatest, and the right extremity of the cavity, from which the heart was farthest removed, was obviously the part that could be penetrated with the greatest safety.

By July 22 the pericardium was again as full as ever, and the general symptoms distressing. An incision was made where the punctures had been, and a tube was put in. This aspiration was followed by some faintness, but subsequently by great relief. After some temporary drawbacks, and three subsequent aspirations of the pleura recovery became complete.

Mr. Parker related a case of extensive pyopericarditis (associated with osteo-myelitis of the tibia) which he had freely incised. Death occurred while irrigation was being practiced to wash out the thick membraniform pus with which the pericardium was distended. As to the best place for the puncture, or opening, Mr. Parker recommended the 4th left interspace, close to the sternum.

Dr. Dickinson's rules for puncture are: 1. Make sure that the pericardium contains fluid. 2. Make out where the heart is. 3. Keep clear of it.—*British Medical Journal*, Dec. 1, 1888.

---

***Hæmatoma of the Ear in Football Players.***—Mr. F. A. Southam says that the traumatic variety of hæmatoma auris is not infrequently met with in those who take part in the Rugby game of football. It may be caused by hard hits on the ear, or in a "tight scrimmage" by the pressure and friction exerted by the arms and bodies of the other forward players on the ears and side of the head, when the latter is bent downwards and forwards. Possibly a predisposing factor is the congested condition of the vessels of the head and neck, the result of severe straining efforts on the part of the player. The affection has become so frequent with the players of the Rugby game that many of the forward players wear a tight-fitting cap with lateral flaps to protect the ears.

In most cases the effusion into the auricle of the ear is slight, and it becomes absorbed spontaneously in a few days. But it is not very uncommon for the extravasated blood, probably mingled with some inflammatory exudation, to become organized and develop into fibrous tissue. This is more likely to occur if the person continues to play, paying no attention to the swelling of the ear. The consequence is, the upper portion of the auricle, the part usually affected, remains thickened and indurated, eventually assuming an irregular shrivelled-up appearance, owing to the contraction that takes place in the newly formed connective tissue. In some cases suppuration occurs at the site of the injury, and deformity occurs after the pus has been evacuated and healing has taken place.—*British Medical Journal*, Dec. 8, 1888.

---

***Bavarian Plaster Jacket for Fractured Spine.***—Mr. F. R. Humphreys says: In a recent case of fracture of the mid-dorsal region of the spine, the patient becoming unmanageable, it was determined to put her up in a plaster jacket. On the third day after the accident this was done, with the assistance of two nurses, as follows:

We took two pieces of house flannel, one $2\frac{1}{2}$ yards long, $\frac{3}{4}$ of a yard wide; the other about 2 inches less in width, and 40 inches in length. We passed the longer piece under the patient in the same way as a draw-sheet; it reached from the acromial process to the lower part of the sacrum. The two sides were now brought together in the middle line in front, and sewn as close to the skin as possible. The axillary portion was split, the edges pared, and shoulder straps attached. Next, the extra pieces of flannel in front were split in about 8 pieces, nearly down to the stitching, and the alternate pieces tied together round a clothes-prop, cut about 2 inches shorter than the bed, so that it could be lifted straight up without knocking it against the bed. The straps were carefully tied, so as for all of them to be of the same tension when the weight came on them, and the patient was then lifted clear off the bed without pain or inconvenience. The lower limbs and the head were supported by a towel tied around the pole, which was then braced up to another one that had been previously placed on the head and foot rails.

The second piece of flannel was then dipped in the plaster mixture and applied to the first piece, muslin bandages wound round the whole, passing through the spaces left in front by the alternate strips of flannel alone being tied. It is advisable to sew the shoulder straps on to the back part before placing this under the patient. —*British Medical Journal*, Dec. 1, 1888.

***Codeine as a Substitute for Morphine.***—Dr. Fischer, of Kreuzlingen, writes to the *Correspondenzbl. für Schweizer Aerzte* that for the last five years he has been using codeine extensively in cases in which morphine is indicated internally. A dose of 0.025 or 0.03 grams of codeine is said to be physiologically equivalent to 0.01 of morphine. Fischer gives codeine internally in powder, but when its bitter taste is objectionable he gives it in a mixture. It may be used externally in the form of suppositories, salves, inhalations, etc. Fischer warns against the rather common idea that the narcotic action of codeine is weak and unreliable. When genuine it has a most marked and constant anodyne and hypnotic action. It never causes bad secondary symptoms, even when given in full doses (as much as 3 centig.) three or four times daily for many successive days. Fischer states that codeine continues to produce its full physiological effects without an increase in the dose being required. The patient does not acquire a codeine habit, nor a craving for the drug. It is especially valuable for the agonizing cough in pulmonary phthisis or bronchitis, and as a hypnotic in all cases of insomnia, except that due to very severe pain.

***Treatment of Furuncle of the Ear.***—Lœwenberg (*Transactions Ninth International Medical Congress*), believing it now demonstrated that furunculosis is caused by the introduction into the sebaceous glands of the staphylococcus aureus, the staphylococcus albus, or the S. citreus, or two or all three, rejects cataplasms and all emollients, using antiseptics only. He uses with great success instillations into the external auditory canal of a saturated or concentrated solution of boric acid; 20 grams of boric acid to 100 of absolute alcohol. When the furuncle is far advanced, and there is no hope of resolution, it is opened by means of the galvano-cautery wire, or the epidermis is scraped off and the above-mentioned mixture instilled. When taken in time they are often aborted, and in all cases they disappear more rapidly and are less painful, especially when cocaine is added to the mixture.

Having noticed the frequency of furuncles of the ear after the removal of plugs of cerumen Lœwenberg now uses antiseptic solutions only for their removal, and they no longer occur. Prophylactic instillations of the solution act well in women troubled by furuncles at the menstrual epochs, and persons who have them at certain seasons of the year.

***The Gastric Juice in Febrile Diseases.***—Dr. Anton Gluzinski, of Cracow, after some extended experiments on this subject, draws the following conclusions:

In regard to the acute infectious diseases:

1. During the whole course of the fever (except in the final stage of typhoid fever) the gastric juice contains no hydrochloric acid.

2. The gastric juice digests neither in the organism—since it contains no peptone—nor outside the organism.

3. This gastric juice digests very well, artificially, after the addition of the proper quantity of hydrochloric acid, which shows that it contains pepsin, and that the impossibility of digestion is due solely to the absence of hydrochloric acid.

4. With the disappearance of the fever, or somewhat later, the gastric juice becomes capable of digestion both within and outside the organism.

In regard to the chronic febrile diseases, normal, digesting gastric juice exists during the fever.

Other things being equal, the nature of the gastric juice in febrile diseases is influenced more by the nature of the injection than by the high temperature. The possibility of a secretion of active gastric juice in chronic febrile diseases increases the hope of good results from forced feeding. *Deutsches Archiv f. Klin. Med.*, Bd. 42, Hft. 5.

---

**Creasote in Pulmonary Phthisis.**—Dr. Beverley Robinson has an interesting communication on this subject in the January, 1889, number of the *American Journal of the Medical Sciences.* He began to use the drug in pulmonary phthisis soon after the publication, in 1877, of an article by Bouchard and Gimbert on its beneficial effects in consumption. He calls attention to the fact that what is called "commercial creasote," obtained from the distillation of coal-tar oil, and having neither the color, the odor, nor the chemical properties of *wood* creasote, should not be used. The best creasote is that obtained from the distillation of *beechwood-tar.* Possibly many of the cases in which creasote has proved inert were cases in which the coal-tar product was used.[1]

As to whether creasote interferes with the bacilli locally, or through the circulation in virtue of its antiseptic properties, or whether, in addition to its promotion of sclerosis, it merely favors general nutrition whilst acting happily upon secondary, though important symptoms, Dr. Robinson is not prepared to affirm, but he is inclined to accept the latter rather than the former theory.

At the beginning of treatment creasote should be taken in small or moderate doses, which should be continued a long time, or only gradually increased. The daily amount prescribed by Dr. Robinson for adults has varied usually from 3 to 6 minims, and continued frequently many months without increase, or interruption, or any evidence of intolerance. The ordinary dose of half a minim is repeated every two or three hours. It is given with whisky and glycerine, according to the following formula:

Creasote (beechwood) ...... ♏vj
Glyercini .................... ℥i
Spts. frumenti .............. ℥ij
S. as directed. M.

In hospital practice, for convenience' sake, or rather so as to give the patient a sufficient supply of medicine to last till his next visit, he prescribes teaspoonful doses, each teaspoonful containing one minim of creasote, and to it are added two teaspoonfuls of water, which prevents irritation of the throat in swallowing the dose, and obviates irritation of the stomach in some cases. The dose is ordered every three hours, so that if taken with absolute regularity the patient gets eight minims of creasote in twenty-four hours. The whisky and glycerine should be perfectly pure, Price's or Bowers' glycerine being probably the best.

Dr. Robinson has frequently prescribed creasote in gelatine capsules combined with cod-liver oil. Each capsule contains about a minim of creasote. They should be taken fifteen or twenty minutes after meals. Two or three doses mark the limit of stomach toleration ordinarily, and in only one or two cases has he been able to increase this number without causing digestive disturbances. This method of administering creasote appears, therefore, to be inférior to the whisky and glycerine mixture. If creasote be administered in cod-liver oil, the amount of oil must be at least one drachm to the minim of creasote, in order to obtain a proper dilution of the drug. Otherwise, if cod-liver oil be indicated it is best to give it separately.

In a large proportion of cases of phthisis that he has treated during the last year or two (and in all of the cases herein analyzed), whilst creasote was taken internally, Dr. Robinson also used antiseptic inhalations by means of the perforated zinc inhaler. As a rule, in the beginning

---

[1] Dr. Robinson says he is credibly informed that the only creasote in the market that responds favorably to all or most of the tests of absolute purity is that of T. Morson & Son, an English product, and that of Merck, of Darmstadt.

the inhaler was worn during fifteen or twenty minutes every three hours, and from ten to twenty drops of the inhaling fluid were poured on the sponge of the inhaler at least three times in 24 hours.

The inhaling fluids most frequently used were:

1. Iodoformi.............gr. xxiv

   Creasoti..................♏iv

   Ol. eucalypti.............♏viij

   Chloroformi............♏xlviij

   Alcoholic ætheris q. s.......℥ss. M.

2. Tinc. iodi ætherealis..........

   Acidi carbolici...........āā ʒij

   Creasoti....................ʒj

   Sp. vini rect............ad. ℥j M.

3. Creasoti....................ʒi

   Alcoholis...............ad. ℥ss M.

The total number of cases to which Dr. Robinson gave creasote mixture and creasote inhalations (simple or compound), were 143 at the New York Hospital, 19 in private practice, and about 150 at St. Luke's Hospital. Of the 143 cases seen at the New York Hospital, 51 were in the first stage of the disease; 18 in the second stage; 18 in the third stage; also 4 cases of laryngeal phthisis, 1 case of fibroid phthisis, and 1 case of acute phthisis; leaving 50 cases in which the stage of the disease is not mentioned. Of the 143 cases, 54 were females and 89 males. Of the 93 cases in which the stage and nature of the disease were mentioned, the duration of treatment in 66 cases (47 hospital and 19 private) varied from one week to two years, eleven months and a half; 45 of these were males and 21 females; 37 cases were affected with the first stage in a manifest manner; in 3 cases the physical signs were doubtful or negative; in 6 there was an evident second stage; and in 1 it was questionable whether the disease had advanced so far as the second stage; in 11 cases the disease had reached the third stage.

In cases of the first stage, 24 had their cough improved, sometimes very much, sometimes only a little; in 3 cases the cough did not improve; in 10 cases the cough was cured. In several cases in which the cough was improved, the sleep was quieter, and previous insomnia evidently depended largely upon cough and expectoration; in a few cases, even though the cough improved, the sleeplessness did not improve, and was evidently independent of the cough. In 3 cases, at the second stage, cough improved either slightly or very much. In the other cases it remained stationary; in no case did it increase. In 6 cases, at the third stage the cough improved notably in 4; in 1 the improvement was very great; in 1 it became worse.

As regards *night-sweats* in the first stage, 8 cases were cured, 4 improved, 3 remained stationary; in 1 case they increased; in 6 cases the patients never suffered from them; in 15 cases no mention is made of the symptom. At the second stage 1 was cured, 1 remained stationary. At the third stage 1 case was cured, 2 improved (1 greatly), 1 patient had no night-sweats, and in 7 cases no mention was made of them.

In the majority of cases in which there was dyspnœa, this was improved or cured under the creasote. The same is true in regard to the sputa and the appetite.

The effect on the weight was very noticeable in many cases. Two, three, and four pounds increase was quite common. One patient gained three pounds in six weeks. Most of the cases gained weight; in only 6 was there loss of weight.

As regards hæmoptysis, it was seen that whilst creasote may not, except to a very limited extent, control pulmonary hæmorrhage, it does not promote nor occasion it, and may be given with perfect safety to patients liable to hæmorrhage, and during the period that they actually occur.

In regard to the effect on the temperature, Dr. Robinson says it is fair to assume that in creasote we have an antithermic agent of no mean value in the treatment of phthisis.

Dr. Robinson concludes his paper by saying that he is convinced that we have in beech-wood creasote a remedy of great value in the treatment of pulmonary phthisis, particularly during the first stage. Not only does it lessen or cure cough, diminish, favorably change, and occasionally stop sputa, relieve dyspnœa in many instances; it also often increases appetite, promotes nutrition, and arrests night-sweats. It does not occasion hæmoptysis, and rarely causes disturbance of the stomach or bowels, except in cases in which it is given in too large doses. It is certainly an unobjectionable medicament from any point of view. It is easy of administration; it is adapted to the majority of sufferers from pulmonary phthisis everywhere; it may be used with some advantage at all stages of this disease, even the most advanced, and in my experience it has proved itself superior to any other medicament with which I am familiar.

---

***Puerperal Insanity of Septic Origin.***—At the meeting of the Medical Society of London, on Dec. 3, Dr. Savage read a paper on this subject, in which he insisted upon the frequent correlation of puerperal fever with puerperal mania, The ordinary form of puerperal insanity, he said, was quite common, and from 8 to 10 per cent. of all cases met with in hospital practice were due to this cause. When it was considered that a large proportion of the cases were treated at home, the total would seem to be somewhat formidable. Puerperal insanity was less curable, he said, than it was generally believed to be. About 5 per cent. of the acute cases proved fatal, and no less than 20 per cent. remained permanently afflicted. When of septic origin the malady most frequently took on the acutely maniacal form, and most of the fatal cases were of this kind. He pointed out that neurotic persons, curiously enough, seemed more liable to septic changes, the determining causes being various. He related the cases of three sisters, all of whom fell victims to the same form of septic mania at intervals of some months.

Dr. Barnes said that obstetricians saw the beginnings, but missed the sequel. Heredity, he said, was the great predisposing cause. As to the evocative causes—blood diseases and such like—he said that Dr. Savage had not drawn a line of demarcation between the mental disturbance occurring during pregnancy, and that which broke out during lactation. It was very important to distinguish between them. Albuminuria was common. The patients had a condition of high vascular tension to which the albuminuria was often due. Then there followed a stage of depression, when the septic symptoms became more marked. In cases that he had seen after labor, anæmia was marked, as was toxæmia from other causes. He said that the liver of a puerperal woman was always fatty, thus increasing the difficulty of disposing of the post-puerperal metabolic products. Emotional shock was a powerful influence in the production of sepsis. Exhaustion was also a factor. He had seen insanity cured by replacement of a displaced uterus.

Mr. Knowsley Thornton said he had had several cases of marked insanity after important abdominal operations. He suggested that the marked acceleration in the pulse, which was considered of such ill-omen in these cases, might be due to septic causes, the other symptoms being masked by the mental condition. He explained the albuminuria, which was often present, to be due to the acute congestion of the kidneys, caused by the elimination of the poison.

Dr. Symes Thompson said there could be no doubt that the susceptibility of puerperal women was largely due to the prostration. Under such circumstances sepsis was more readily induced.

Dr. Savage said that statistics seemed to point to a preponderance of cases among the upper classes. He said there was no more danger to reason from puerperal mania than from the delirium of fever. He preferred a tonic treatment.—*British Medical Journal*, Dec. 8, 1888.

---

***Strychnine as an Antidote in Narcotic Poisoning.***—Dr. G. A. Gibson calls attention to the treatment of the effects of narcotic poisons by using strychnine to avert the paralyzing action of such drugs on the medullary centres. Narcotic drugs in lethal doses cause death by paralyzing the respiratory centre. In the cases of narcotic poisoning that have come under Dr. Gibson's notice during the last three years strychnine has been used hypodermically, when there was any irregularity or interruption of the breathing that appeared to threaten a failure of the respiratory centre. The effect of the drug is immediately shown by the increased rate, more regular rhythm, and greater depth of the respirations, and even in cases in which the breathing had ceased, it began again after the administration of strychnine.

In the treatment of poisoning by narcotics, says Dr. Gibson, two things should be studiously avoided: 1. Making the patient walk, and flogging him, to keep him awake. Such treatment has a tendency to exhaust the vital powers. 2. The use of alcoholic stimulants, which aid the action of narcotics.

The patient should be kept in the horizontal position, the respiration is to be carefully watched, and should there be the least sign of irregularity, or shallowness, or inequality in the breathing, gr. $\frac{1}{150}$ or $\frac{1}{50}$, according to the age of the patient, of sulphate of strychnine should be given subcutaneously, and may be repeated at intervals of an hour two or three times. If, notwithstanding this, respiration becomes very feeble, or ceases entirely, artificial respiration must be begun promptly. If the circulation threaten to fail in consequence of the poison affecting the motor mechanism, or of spasm of the arterioles caused by deficient oxygenation of the blood, it should receive prompt attention. Strychnine may be used as a stimulant to the motor centres of the heart, and may be aided by the use of ammonia or ether. If artificial respiration has been thoroughly performed, there should be no spasm of the arterioles; but should there be any, nitrate of amyl must be used. Strychnine may be used also in cases of threatened failure of the respiratory centre, caused by the general anæsthetics. — *The Practitioner*, December, 1888.

---

***Elastic Pressure in Mammary Inflammation.***—At the meeting of the Royal Academy of Medicine in Ireland, Section of Obstetrics, on Nov. 23, Dr. Andrew Horne read a paper on this subject. He said that inflammation of the breast was almost always the result of infectious material gaining entrance through fissures and cracks of the nipple. The treatment advocated was to envelop the breast in a layer of absorbent cotton. Having first painted the breast with a 5 per cent. solution of oleate of mercury and morphine, an elastic web bandage is used to make gradual and equable pressure over the inflamed gland, thereby securing the most perfect rest possible.

Dr. Macon said that he had long used compression of the breast in certain cases, though he did not regard it as suitable where there was suppuration; but he could recommend Dr. Horne's method, even where there was suppuration, as strongly as in other cases. It gave great relief to the patient. In certain cases it would permit the woman to go on nursing, which, in case of the poor, was a great advantage. It seemed specially applicable in the troublesome form of the affection in which, though the breast was enormously distended, and the skin quite tight, not a drop of milk would flow.

Dr. Smyly said he used to employ Martin's bandages, but he believed Dr. Horne's arrangement to be much better. One of its best features was the covering up of the breast, thus preventing external infection. He believed one of the causes of mammary inflammation was the handling of cracked nipples by the midwife, whose fingers are not always aseptic.

Dr. Horne said that it was most difficult to put on elastic plasters so as to get even pressure, and they are apt to cause an eczematous eruption on a tender skin. The Martin's bandage causes an uncomfortable heat, which the elastic web bandage does not.—*British Medical Journal*, Dec. 8, 1888.

---

***Quantitative Estimation of Uric Acid.***—Dr. August Herrmann has recently reported the results of some experiments with Haycraft's method of estimating uric acid, which is based on the fact that nitrate of silver is very insoluble in water and ammonia. The urine is first treated with bi-carbonate of soda, is then made alkaline, and ammoniacal solution of silver is added. The precipitated urate of silver is collected on an asbestos filter, and washed. It is then treated with nitric acid, by which it is dissolved, and in the filtrate thus obtained the silver is estimated by Volhard's method; and from the amount of silver found the amount of uric acid can be calculated. Dr. Herrmann has slightly modified this method: he used a large quantity of urine, and added magnesia in addition to bicarbonate of soda; he treated the wash-water with hydrochloric acid instead of a solution of common salt. He compares Haycraft's method with Ludwig's method: in the former two analyses gave the same result; in the latter there was a difference of + or — 0.00038 grain; in the former the results were somewhat higher, on an average 0.0029 per cent., probably due to a precipitation of xanthin by the silver. The constant error in both, estimated by using pure uric acid, is — 2 per cent. He found that sugar and albumin interfere very slightly with Haycraft's method, and need not be separated before estimation. Though this method gives, as compared with Ludwig's, too high a result, yet for comparative and clinical purposes, where a knowledge of the variation in the amount of urea present is sought, it is to be preferred on account of its practicability and the short time required for its performance.

---

***Treatment of Syphilis.***—Professor Edward Lang considers metallic mercury the sovereign remedy in the treatment of syphilis. Internally he recommends:

Lanolin mercurial ointment (50 per cent.)..................3 grams
Milk sugar................7 "

Make 60 pills. Take from 4 to 6 pills a day.

Lang recommends Unna's mercurial plaster, especially in the treatment of syphilis in children. A part of the surface of the body should be covered for from 4 to 6 days, and after this another part of the body.

In order to prevent eczema, gray plaster should be used, made as follows:

Olei crudi................180
Plumb. oxydat.............100
Hydrargyri................ 60

For subcutaneous injection Lang thinks gray oil best, prepared as follows: 5 grams of lanolin are dissolved in 20 grams of chloroform, and 10 grams of mercury should be rubbed up with this until the chloroform disappears.

Lang recommends the following formula for using calomel:

Calomel
Lanolin āā................2.7
Olive oil ................4.6

Lang considers subcutaneous injections of calomel and gray oil better than inunctions.—*Internat. Klin. Rundschau*, No. 51, 1888.

***Water in Obesity***—LORENZEN, of Erlangen, has made some experiments on himself in regard to the influence of liquids on obesity. For a period of nine years he drank daily a large quantity of Erlanger beer. For four years of this period the quantity consumed daily amounted to 10 litres, or about 22 pounds; during the remainder of the period the quantity was between 5 and 7 litres, with 1 litre of wine. In this way he succeeded in increasing his body weight by 78 pounds. On shutting off the liquids his weight fell 14 pounds in 7 days; but when more water was taken, even without alcohol, the weight again increased. Within five weeks he reduced himself 28 pounds. Similar experiments, carried out on some of his heavy colleagues, gave similar results. Lorenzen explains the disappearance of fat, when fluids are withheld, on the hypothesis that the cells whose province it is to decompose albumen, when a large quantity of fluid is taken, now expend a part of their energy in the combustion of fat.—*Medical Press—The Sanitarian.*

---

***Jumbul in Glycosuria.***—MR. GEORGE MAHOMED, of Bournemouth, records his first experience with jumbul (or jambul), in diabetes. The patient was a man about 60 years of age, and had had diabetes about nine months, to his own knowledge. The urine had a specific gravity of 1020, and contained a considerable amount of sugar. He was put on *perles* of jumbul, a two-grain *perle* three times a day. At the end of a week the urine contained no sugar. On the drug being discontinued the sugar reappeared, and disappeared again when the jumbul was resumed. As the patient experienced considerable temporary depression, the quantity of jumbul was diminished to two *perles* a day, on alternate days. Nearly four months after the first visit the urine still contained no sugar, and after more than a year the patient believes that he is cured.—*Practitioner*, December, 1888.

***Yeast in Infectious Diseases.***—In a recent number of the *Deutsche Medicinische Wochenschrift* attention is drawn to the use of yeast in the treatment of certain diseases. In an epidemic of scarlatina and diphtheria it was employed to the exclusion of every other medicament. The scarlatina cases developed without complication, and with an ordinary amount of fever, while in the diphtheria cases the false membrane rapidly separated, and lesions of the heart and paralysis of the muscles did not supervene. The mean dose was one teaspoonful every hour. Gargles of yeast, also, in the proportion of one part of yeast to five of water, were found to be of service. The yeast does not cause diarrhœa; in fact, it may be recommended in the diarrhœa of infants.—*Medical Press and Circular*, Nov. 14, 1888.

---

***Treatment of Typhlitis.***—BOUCHARD recommends the following plan of treating typhlitis: 1. Alleviate the pain, either by injections of morphine or the application of belladona ointment under a hot poultice. 2. Wash out the large intestine by copious hot enemata, consisting of at least a quart of hot water in which ℥jss of borate of sodium is dissolved, or to which two or three teaspoonfuls of a mixture of equal parts of tincture of benzoin and spirit of camphor is added. 3. The patient must be kept absolutely quiet. 4. Only the mildest kind of purgatives, if any at all, should be used. 5. Food should consist of milk diluted with alkaline waters, with the yolk of an egg at a later period.—*France Médicale*, Sept., 1888.

---

***Antipyrin in Chorea.***—HAJOS injects hypodermically a 50 per cent. solution of antipyrin in chorea, beginning with ♏vijss and doubling the dose daily until gtt. 60 are given at one injection. He uses antipyrin also in intercostal neuralgia.

